# Children’s understanding and consent to heart surgery: Multidisciplinary teamwork and moral experiences

**DOI:** 10.1177/13674935221100419

**Published:** 2022-09-27

**Authors:** Priscilla Alderson, Hannah Bellsham-Revell, Nathalie Dedieu, Liz King, Rosa Mendizabal, Katy Sutcliffe

**Affiliations:** 1 Social Research Institute, 4919 University College London, London, UK; 2 Evelina Children’s Hospital, 8945 Guy’s and St Thomas NHS Trust, London, UK; 3 4956 Great Ormond Street Hospital NHS Trust, London, UK; 4 School of Health, Wellbeing and Social Care Open University, Milton Keynes, UK

**Keywords:** Childhood illness, Children’s rights, consent, critical thinking, ethics

## Abstract

Mainstream law and ethics literature on consent to children’s surgery contrasts with moral experiences of children and adults observed in two heart surgery centres. Research interviews were conducted with 45 practitioners and related experts, and with 16 families of children aged 6 to 15, admitted for non-urgent surgery, as well as an online survey. Thematic data analysis was informed by critical realism and childhood studies.

Impersonal adult-centric mainstream literature assumes young children cannot consent. It is based on dichotomies: adult/child, competent/incompetent, respect or protect children, inform or distract them, use time swiftly or flexibly, verbal/non-verbal communication, respect or control children and reason/emotion.

Through their moral experiences, adults and children resolve these seeming dichotomies. Through understanding young children’s reasoning and emotions about complex distressing decisions related to heart surgery, adults share knowledge, control, trust and respect with them. They see children’s consent or refusal before non-urgent surgery as a shared personal moral experience within the child’s life course, beyond mere legal compliance. Adults help children to understand and ‘want’ the surgery that offers things they value: better health or to ‘be more like their friends’. If children are not convinced, sometimes surgery is postponed or occasionally cancelled.

## Introduction

Mainstream literature on the law and ethics of consent to children’s surgery clearly differs from current practices and moral experiences of children and adults observed and reported in two heart surgery centres. Law and literature are mainly concerned with parents’ consent (Chotai et al., 2017). ‘Assent’ is often the preferred term with children. Yet this US concept conflicts with English Gillick law (Biggs, 2009; Alderson, 2012) and is ‘incoherent and wrong’ (Baines, 2011:960). Assent has gaps, such as largely omitting dissent (Montreuil et al., 2020). This denies the moral theory of consent as a free choice to safeguard personal integrity, agreed internationally for decades (Nuremberg Code, 1947; WMA, 1964). Even babies show a rudimentary sense of personal integrity when they enjoy cooperating but become very upset about enforced interventions (Gopnik, 2009).

Consent forms are designed to prevent costly complaints or litigation by patients, and the signature must be legally valid, written by an adult with parental responsibility, or a Gillick competent child (Brazier and Cave, 2016; Gillick [1985]) (or in the US by a mature minor). A Gillick competent child ‘achieves a sufficient understanding and intelligence to enable him or her to understand fully what is proposed’ and ‘sufficient discretion to enable him or her to make a wise choice in his or her best interests’ (Gillick [1985]). There is no minimum age of competence though experts mainly assume that competence begins around 12 years or above, and that legal minors under-18 ‘cannot refuse’ recommended major treatment (Brazier and Cave, 2016:466; Griffith and Dowie, 2019).

The few studies of children’s own experiences find that many younger children want to share in deciding about their medical care with adults (Alderson, 1993; Alderson et al., 2006; Alderson et al., 2022a, 2022b). Coyne et al. (2014:273) found that children ‘were generally involved in minor decisions’ about their cancer care by practitioners whose aims were ‘gaining their cooperation, making treatment more palatable, giving back a sense of control and building trusting relationships’. Yet ‘some adolescents were aware that choices were not “real” decisions since they were not allowed to refuse and expressed feelings of frustration.’ Young children can be highly knowledgeable about chronic illness (Coyne, 2020). Formal shared decision-making tools can ‘increase the children’s knowledge and satisfaction and reduce decisional conflicts’ (Wijngaarde et al., 2021:2345).

Children seen as not yet competent to consent have no legal right to be informed, to have their views respected, and not to be coerced or deceived (Wellesley et al., 2021). The mainstream literature endorses enforced treatment but overlooks its problems (Lauro, 2018). However, in the nursing literature, there is concern about restraint (Bray et al., 2015, 2019; Nielson et al., 2021). Children are ‘held still for clinical procedures quite often (48%) or very often (33%)’ while procedures are enforced (Bray et al., 2018). With adult patients, this would be illegal assault and with children should only be a last resort (British Medical Association, 2016).

Moral values and actions have been summarised into four principles, which influence medical ethics (Beauchamp and Childress, 2019). These principles have helped greatly to increase respectful care for adult patients, but they can be less helpful for children in the following ways:

• Justice may be seen as equal care for all and fairly distributed resources. This is complicated when children have varying needs. Many depend on extra resources, such as time-consuming explanations before surgery. These can conflict with efficient use of scarce time and resources and may cause delays that harm other patients.
• Respect for autonomy may apply to rational independent (adult) patients. Yet if children are seen as too immature and dependent to be autonomous, they lose binding legal rights, although these exist in international guiding law: to be informed and involved in decision-making and to be protected from (what some may feel is) ‘cruel, inhuman or degrading treatment’ (UN, 1989, Articles 12, 13, 37).
• Doing good is often taken to mean defending the child’s best interests (UN, 1989, Article 3), as defined by adults, not by the child (Goold et al., 2019).
• Do no harm may then be interpreted as avoiding neglect or omissions that could follow if a child’s refusal of clinical interventions is respected. This can justify ignoring children’s views and protests, but well-intentioned enforcing of interventions can distress practitioners and children (Bray et al., 2018; Miller, 1983).

The general approach to morality and bioethics by Beauchamp and Childress (2019) has been criticised as too concerned with an imagined rational emotion-free (adult) autonomy, too generalised, impersonal and remote from everyday life. For example, Mary Midgley (2001:59, 48) contended that moral philosophy should be more concerned with how ‘ordinary life contains a great deal of hard moral thinking’ while we cope with life as ‘a flood of jumbled material that needs to be picked over and sorted out by endless imaginative work’. Critical realist Andrew Sayer (2011:13) advocated thinking ‘about social life from the inside, as participants and agents as well as from the outside as spectators’. Both authors supported interdisciplinary research about how we are rational animals who suffer and flourish within networks of caring relationships, and understanding through our embodied emotions as well as through reasoning. Nurses have long analysed tensions between the detached ethic of justice, favoured in the consent literature, and the engaged ethic of care (Wood, 2011), which influences nursing ethics and this paper. Although doctors carry legal responsibility for respecting consent to surgery, nurses inform and support parents and children, as skilled communicators and patient advocates (Menendez, 2013; Farmer and Lundy, 2017; Bray et al., 2018). Research on children’s consent to surgery in two London paediatric cardiology departments found great differences between approaches to morality in mainstream law and ethics literature versus daily life in these hospitals (UCL, 2021; Alderson et al., 2022a; 2022b). This paper reports the research and considers ‘from the inside’ the moral experiences of young patients and the adults caring for them.

Critical realism’s three levels of reality also inform this paper. The *empirical* level involves thinking, the *actual* level is about being and doing and the mainly unseen *real* level is about causes, why events occur or decisions are made (Bhaskar, 2016; Alderson, 2021). Table 1 shows how consent works at three levels.

**Table 1.** Three levels of reality and moral experiences of consent and refusal.

Level	Reality exists in	Examples of moral experiences of consent/refusal
**Empirical**	Thinking/feeling: Ideas, explanations, descriptions, memories, statistics, facts, images and perceptions	Patients’ understanding of informed consent
**Actual**	Being/doing: Events, relations, structures, interactions, heart conditions, interventions and outcomes	Children’s active cooperation or resistance and adults’ reactions
**Real**	Mainly unseen causal powers; policy and economics of paediatric cardiology services and personal motives, hope and aims	Motive-led willing consent: children’s needs, hopes, trust and willing consent, or fear and mistrust; practitioners’ motives to promote health and high standards, and respect patients; power, control over decisions

The Nursing and Midwifery Council Code (2018), for example, starts with the *real*, mainly unseen, powerfully motivational spirit of professional standards: ‘treat people as individuals and uphold their dignity... with kindness, respect and compassion... work in partnership...respect the contribution that people can make to their own health and wellbeing...[and their] right to accept or refuse care and treatment’. No age limits are mentioned so that children seem to be as much involved and respected as adults. Later sections of the Code cover *actual* and *empirical* levels, such as specific practical details on protecting safety and privacy.

Mainstream consent law and literature pose dichotomies: adult/child and competent/incompetent (Brazier and Cave, 2016:466; Griffith and Dowie, 2019). These dichotomies can force choices, either to respect children or protect them by forcing them to undergo interventions. Later sections will show how dichotomies can be resolved in the search for truth (Bhaskar 2008).

## Aims

The aims of this article are to report current moral experiences of children and adults before children’s non-urgent heart surgery and to show how these differ from assumptions in mainstream law and ethics literature.

### Research methods

Ethics approval was granted by NHS Health Research Authority (19/LO/0073), local Research Ethics Committee (ID 248332) and HRA Confidential Advisory Group (19/CAG/0148). This qualitative research reviewed the law, ethics and healthcare literature on consent to children’s major treatment. Our ethnography included observations of wards, clinics and interdisciplinary meetings, and face-to-face interviews in two paediatric cardiology departments from October 2019. With their written informed consent, adults and children gave audio-recorded semi-structured interviews with open-ended questions. There was a purposive sample of 45 senior hospital staff and related experts, listed in Table 2.

**Table 2.** The 45 professional interviewees.

Number in each speciality^a^	Speciality
6	Nurses
26	Consultants – congenital heart disease cardiologists, surgeons, anaesthetists, paediatricians, intensivists and a palliative care specialist
2	Play specialists
4	Chaplains
5	Psychologists
1	Transplant team social worker
1	Children’s heart charity information staff
3	Lawyers
1	Mediator
8	Members of ethics committees
4	Members of a hospital directorate

^a^Some of the 45 interviewees had two or more present or previous roles.

Sixteen families were observed before and after elective heart surgery. Thirteen adults in 12 of the families were interviewed shortly after the operation and some months later; only six of the children aged 6 to 16 gave interviews.

We hoped to meet many more children, but after February 2020, because of COVID-19, elective surgery was cancelled, our observations and direct contact with participants ended and interviews were held by telephone or online up to April 2021. Three children’s heart charities conducted our online survey with their members; 15 children and 23 parents replied. Two charities conducted discussions based on our interview questions by telephone or email with 16 children and young people aged 6 to 17.

Encrypted interview audio-recordings were professionally transcribed and anonymised. Thematic data analysis was informed by critical realism and grounded theory (Bryant and Charmaz, 2010), and the sociology of childhood, which respects children’s agency (Alderson, 2013; James and Prout, 2015; Alderson, 2016). All cited participants are anonymous and non-identifiable; practitioners are referred to by their profession, and children by their pseudonym.

### Findings: Moral experiences and uncertainties of practitioners and families in the two hospitals

The next sections report moral experiences of the multidisciplinary paediatric cardiac teams and families working together to resolve moral uncertainties.

### To protect or respect children

When children are admitted for heart surgery, many parents feel unable or unwilling to inform them, saying their children should not be alarmed by details about the nature and purpose of the surgery, details central to informed consent. From experience, practitioners said in interviews that they know uninformed, unprepared children tend to resist interventions. A surgeon avoided force and ‘fights’

*because it’s a bad situation…you’ll get bad results by a bad practice, you know, there’s a physiological reason not to do it and then there’s the greater psychological reason why you shouldn’t be doing this.*

An anaesthetist said,

*we often see children who are terrified, having previously been held down for induction of anaesthesia by teams who did not mean to cause harm, but were just trying to get a procedure done. Rebuilding lost trust is a long and difficult process.*

These practitioners therefore combine protection with respect by helping children to understand and actively cooperate with their care.

Interviewees also spoke of respecting parents by persuading them to allow their child to be informed. A play specialist talked of first listening and understanding parents’ anxieties and then being able to work alongside them, to gain their trust and their consent to prepare the children. She found many parents were relieved to learn how to share information. The mother of Duncan aged 8 said,

*So he’s very aware that he could die and it’s about, you know, preparing yourself for those questions in a way that if my child comes up to me and says, “Mummy am I going to die?” I don’t completely fall apart and think, “Oh my God what am I going to say?”*

Through talking together, parents were more able to both respect and protect their children through advocacy. Gary was also aged 8. His mother said,

*When anything hasn’t been quite right or he hasn’t felt as good as it could be for him, we’ve talked about how we could make it better and then I’ve advocated for him with whoever I needed to do that with in order to make that process a lot easier.*

### To inform or distract

Interviewees were convinced children should be informed as much as they needed and wanted to be, to prepare them to cope with all perioperative procedures. They promoted moral relationships of honesty and trust. They described using distractions, such as toys and jokes, with anxious children, not as alternatives to giving information or to deceive children but to combine distractions with the child’s informed willing cooperation. An anaesthetist considered too much distraction risks reducing

*a child’s ability to engage with the process and develop both their emerging decision-making autonomy, and a sense of responsibility for managing their own health. Distraction is best when a child knows what is actually happening but is actively participating in the distraction in order to help them manage what would otherwise be an unpleasant experience.*

### Time: Efficient systems or flexible personal care

Interviewees critically described examples of adults who believe efficient quick encounters are less upsetting for children, who soon forget them. With interventional catheterisation, when children remain conscious, their cooperation is vital. A cardiologist had

*one catheter patient who refused, an 11 year old with learning difficulties, so borderline Gillick competent, and we did a lot of explaining, therapy and reframing, and we were then able to proceed a few months later - this was a clinically needed procedure, but we decided not to force the issue, and to do it on her terms.*

Time invested in preparing and informing children before surgery helps to prevent delays in the anaesthetic room when non-emergency surgery might be postponed. Anaesthetists said they could more easily make the moral decision to refuse to impose anaesthesia on a protesting child as young as 4 years, and possibly cancel the operation, when they had the support of the multidisciplinary team.

Through meeting their patients regularly for years in clinics, paediatric cardiologists knew them well. They saw the need to invest time in both efficient and flexible personal care: informing and preparing families as much as possible. Young patients are prepared, by encouraging their trust and cooperation, for the later transfer to adult services, when they take on more moral responsibility for their own healthcare.

### Communication: Verbal and non-verbal

Words and verbal records are central to law, ethics and healthcare. Yet moral respect for young patients also includes communicating with them beyond words, in two important ways they value, seldom considered in the law and ethics literature.

First, there are the constant physical interactions between adults and children, affected by adults’ skill and respect and children’s cooperation or resistance. Even babies were soothed by a gentle touch. Practitioners were observed aiming to do things *with* children rather than *to* them: waiting and avoiding force, or offering children a choice between the anaesthetic mask and cannula. A cardiologist respected an 8-year-old girl’s privacy and modesty by drawing the screen round the examination couch and asking her to remove as little clothing as possible.

Second, there are ways of informing children through images, models and toys to prepare them for interventions, encouraging them to play with equipment and dolls with scars and connected to tubes. There were pre-admission tours of the wards and theatre and intensive care areas. When merged with talk and stories, hospital play increases children’s understanding, helps them to feel prepared with some sense of control over potentially distressing events and reduces misunderstandings and avoidable fears. Role play helps children to see that distressing interventions are intended to help and not harm them. A psychologist spoke of 2-year-olds having some idea of being hurt or poorly and needing care ‘that will help you feel better’. Children valued medical information shared with them through drawings: Martha aged 12, through a model of the heart, Gary aged 8 and Duncan aged 8 who has learning difficulties understood more when he was shown how his narrowed valve would be widened by a balloon being inflated inside it.

Nurses and doctors praised the play specialists and psychologists during their interviews and routinely referred resisting children for play therapy. The MRI for Duncan was postponed for a month while through games and other activities, psychologists helped him make sense of the process and overcome his anxiety about memories of his previous surgery. Duncan’s mother said,

*So they break it down into such simple steps he could cope with it.*

Respecting children involves communicating with them in verbal and non-verbal ways they prefer and understand.

### Control: By the adults or by the child

Consent clarifies who has power over the decision and whether power is monopolised during disputes or shared through consensus. Young patients, however, need to know that adults are using their power to help and respect them, not as arbitrary control. Adults could do this formally. Emma aged 11 refused to have an echocardiogram the day before her surgery, and her doctor used another recent one instead. A nurse spoke of nurses spending so much time with the young patients they could ‘be privy’ to children’s doubts about surgery.

*But even things like blowing up a glove and drawing a smiley face on it makes toys out of a glove, even things like that, it just says to them, “I’m your friend, well I’m on your side”.*

When Becky aged 12, who has learning difficulties, was admitted for surgery and refused to take off her coat or sit on the bed, she was referred for five weekly play therapy sessions. The play specialist said:

*Dad kept saying to me, “She’s not going to lie on the bed...She doesn’t like the up and down.” I said, “Dad, what I need you to do is stop being so negative because I don’t think that’s helping. Let’s have a lot of positive vibes around her.”*

A play specialist lay on the bed while Becky pressed the up and down button and ‘thought it was hilarious’. Soon she willingly lay on the bed, and later, she managed to have an echocardiogram. After ‘silly games’ with a monkey doll and the X-ray machine,

*we said, “Becky, all you have to do is hug the X-ray”. We managed to unzip her coat and as she hugged the X-ray, the woman took the picture.’*

When Becky was admitted, they put her own cushion and sheets on the bed. ‘We had a beautiful session...she was more than ready’ for the operation.

By sharing control informally, the adults achieve positive ends through play, negotiation and shared control and decisions. Occasionally, after long negotiations, the refusal of children aged from about 6 years may be accepted as final, for example, if a child still refuses a heart transplant. Interviewees warned that unwilling children may not survive long because daily lifelong follow-up care depends on their active cooperation and consent.

### Reason or emotion

In contrast to the imagined emotion-free rationality noted earlier, valued by many philosophers, interviewees showed how moral experiences of consent involve reason and emotion. Practitioners took all children’s distress and anxiety seriously, as warnings that they needed help. Interviewees spoke of respectfully relieving children’s fears, correcting misunderstandings, and offering reassurance. Among the 16 interviewed families, a quarter of the children were afraid because previous operations had been cancelled, when overbooking and delays are routine. Of the six children interviewed, four worried about memories of the anaesthetic gas and all reported anxieties about pain. Five were afraid they might not ‘get back to normal’ after surgery. Two were anxious about tubes and wires, and one child feared waking up during the operation. Three feared they would die. A children’s heart charity youth officer spoke of children fearing the process whilst wanting the benefits:

*they’re scared of having the transplant, but they’re terrified of not being eligible for the transplant at the same time.*

These worries are likely to be under-reported. Admitting fears can feel shameful and embarrassing, and hard for children to raise, when they tend to protect their parents by not talking of their own anxieties, as practitioners described. Chaplains reported young children confiding in them privately about their fear of dying.

Consent involves emotions of ‘wanting’ the surgery and having moral courage to undertake the harms and risks to gain the hoped-for benefits. It could be easier for children with symptoms to see the need for surgery. Gary aged 8 was having a ‘normal’ life, but his mother said he was becoming

*significantly slower and his sats decreased a lot, he gets tired quicker and he breathes quicker.*

The longing to keep up with their friends helped children to agree to surgery. Many knew they would need elective surgery to prevent or reduce future problems. Yet the decision to operate could still be ‘a big shock’ as Rafe aged 14 said. He felt asymptomatic and was very active in sports, though he agreed with the decision. A few months after surgery, he was pleased and grateful that his sports energy was even better than before his operation.

Understanding the nature and purpose of the surgery helped children to feel willing to consent. A psychologist described shared decision-making when

*parents and children and the team come together, everyone’s got enough information, everyone has an understanding about why this needs to be done.*

Discussing the news that surgery was needed, Kaleb aged 13 said firmly,

*I'm going to go through with it. I'm prepared. I knew that it was going to happen and I'm prepared.*

The necessary supra-regional teamwork to prepare children could include local practitioners. A cardiologist described a child who resisted surgery:

*We actually aborted the procedure and she's now done some work with her local community team and… since she's been home, she's actually been saying, “I want to have the procedure”, so not just that she's not refusing it, she's actually been saying, “Actually yeah, I think I do need it, I think I want it”.*

A nurse said that difficult conversations also took place outside the hospital through cardiac liaison nurses.

*All this back work had been done, all these difficult conversations had been had, tears had been shed. And by the time they came to the ward they were just ready to go or ready for the surgery.*

Parents experienced moral emotions of empathy and distress for their child as they shared the thinking-feeling journey through surgery. Moira aged just 16 had learning difficulties. Her mother said she felt

*shocked. I don’t know why because I’ve always known [she’d need surgery], but for some reason I didn’t think she looked ill enough. She’s had...anaemia and other things, not being very well for about the last year, but I still was shocked and then obviously upset. I’ve been through every emotion possible I think since.*

A psychologist observed,

*So a big part of our work is working with the parents to help them manage their distress or their worry so that they’re then able to be with their child and be kind of more present with their child - which then helps the child feel more calm and supported and safe.*

## Discussion

Mainstream law and ethics literature is mainly silent on young children’s rights to be informed and involved and tends to assume dichotomies of opposing concepts. Young patients may either be respected or else protected with enforced treatment if needed. Anaesthetists may either inform or distract young patients (Litman, 2011; Massie et al., 2021). There are cost-effective efficient quick interventions or slow inefficient ones (Munteanu and Jordan, 2017). Information is verbal not non-verbal. Control over decisions must be held by adults and denied to children in their best interests (Goold et al., 2019; Bridgeman, 2021). ‘Perhaps the most provocative and surprising finding of our review was that interventions [about decisions] rarely targeted patients (i.e., children) but focused mainly on parents’ (Wyatt et al., 2015:577). Consent is rational not emotional. These dichotomies can limit how practitioners think about options for decision-making and care. Yet our findings show that in practice, the options are not really opposed.

Our findings are better supported in the nursing literature: Distraction and hypnotherapy techniques can reduce informed children’s anxiety and pain (Birnie et al., 2018). Nursing research examines how much ‘holding’ and force are necessary and acceptable (Bray et al., 2015, 2018; Lombart et al., 2019, 2020). A ‘clinical pause’ gives professionals ‘time to consider children’s expressed wishes and explore alternative approaches’ (Bray et al., 2019:160). Healthcare professionals who took time to explain and ‘gained permissions (assent from children and/or consent from parents) before procedures were less likely to hold children still for a clinical procedure than those who did not’ (Bray et al., 2018:205). Firmly holding resisting children can cause practitioners to feel moral distress in uncertainty, guilt and upset and in breaching their trust and protective relationships with children (Bray et al., 2019). Lucy Bray and colleagues advise that, ‘Robust evidence, debate and recognition are needed concerning how resistant children are frequently [forcibly] held for non-urgent clinical procedures’ (Bray et al., 2015:157). Partly non-verbal play is also reported to aid recovery and reduce pain, stress and anxiety (Elcock and Shapcott, 2015; Schalkers et al., 2016; Gjærde, et al., 2021; Salmi and Hanson, 2021).

Thematic analysis of the literature, research observations and interviews shows how the processes, when adults inform, prepare, support and respect children before elective heart surgery, are moral experiences for all concerned. Childhood studies theories about experience rather than age influencing the development of children’s competent agency (Alderson, 2013; James and Prout, 2015) are demonstrated in adults’ respect for young children’s decisions about heart surgery. Critical realist analysis that observes processes ‘from the inside’ (Sayer, 2011:13) reveals unseen causal influences of personal motives and values, and moral feelings of hope and fear, central to consent.

To enforce non-urgent heart surgery ‘in the child’s best interests’ may protect the child’s body but not the child’s mind, agency and trusting relationships. Negative coercive power can be changed into positive supportive power (Bhaskar 2008, 2016) when adults share knowledge, power and control with children. This involves questioning received child/adult dichotomies and resolving uncertainties created by dichotomies, showing how conflicting ideas can overlap and interact (Bhaskar, 2008, 2016; Alderson, 2016, 2021). Informed by their experiences of illness and treatment, many children share adult insights and maturity, while adults’ knowledge and agency are limited and fallible, and their autonomy is qualified by their interdependence. ‘Adult’ communication about the nature and purpose of surgery may be combined with play. Protection and respect involve recognition that consent is an emotional journey, which, for example, Becky made, from fear to trust and confidence. Respecting children’s rights to have their views (including protests) heard and to influence decisions about their best interests (UN, 1989, Article 12) takes time but can be cost-effective when it saves many future resistances and delays and promotes cooperation. When practitioners combine detailed information with reassurance, they work at the *empirical* level of words and ideas, in *actual* skilful interactions, and at *real* levels motivated by kindness and respect to encourage children’s trust (Table 1). Sharing information as far as children can and want to understand helps them to cooperate actively with their care when they share the practitioners’ real motives to promote their health and well-being.

Moral experiences of practitioners and families showed the four principles (Beauchamp and Childress 2019) can be more morally child-centred:

• Justice can then mean allotting resources to each according to his or her need.
• Respect for autonomy includes discovering why children are afraid and resistant, respecting their informed moral emotions and helping them to give their informed and willing consent. In rare cases, it means accepting the child’s final refusal.
• Doing good includes working respectfully with children to overcome the false assumed conflict between children’s best interests and their informed views.
• Do no harm means avoiding either enforced treatment or the neglect of withheld treatment and instead negotiating with children who resist and refuse. Rejection of a mask or needle is not mindless but is the child’s instinctive self-preserving avoidance of danger. Effective medical and nursing care is therefore based on helping young children to understand how each procedure serves their safety and best interests and on listening to children and learning from them.

### Study limitations

This small study was of only two exceptional settings, where the large teams had unusual amounts of time, training, skill, funds and resources. The children were also unusually experienced in the dangers of serious illness and high-risk surgery. COVID-19 limited the data collecting, as noted earlier. Interviewees may not be representative and they may have presented over-favourable accounts. Yet their general agreement was supported by our observations.

### Implications for practice

Our sample demonstrates that certain (not all) young children can be moral agents and share in complex high-risk healthcare decisions. This suggests many other children (and adults) do or could benefit from being carefully informed and involved across a wide range of healthcare decision-making including less risky and complex cases. This requires skill, developed through careful training, continuing support from senior staff and managers, committed teamwork, shared moral standards and adequate time and resources.

These children need lifelong cardiac care. There was great concern that some develop bad fearful memories of forced interventions and later opt out of adult services. Practitioners stressed their moral responsibility to sustain these children’s trust in their cardiac care.

## Conclusion

The need for children’s active cooperation, especially with heart transplants, has shown practitioners how young children can understand complex distressing decisions, the advantages of respecting children’s views, and the dangers of overriding them. The key moral challenge then for adults is how they respect children’s informed willing consent or refusal. This involves checking how much children understand and also how well the adults inform and support them.

Whereas the four bioethical principles tend towards impersonal abstract analysis of consent, this study understands consent to be a shared personal moral experience within the child’s life course, beyond mere legal compliance. Practitioners and parents help children to ‘want’ to have surgery which they understand will help them to enjoy things they value: better health or to be more like their friends. Not all children understand the techniques and risks of the surgery as much as adults’ and parents’ consent entails. Yet children can share with adults the ‘real’ level and moral experience of motivated voluntary consent, central to practitioner–patient relationships of mutual trust and respect.
